# Left Atrial Functional and Structural Characteristics in Patients After Total and Bicaval Orthotopic Heart Transplantation

**DOI:** 10.3390/jcm13247643

**Published:** 2024-12-15

**Authors:** Marta Obremska, Roman Przybylski, Mateusz Sokolski, Monika Przewłocka-Kosmala, Mateusz Rakowski, Jakub Ptak, Przemysław Sareło, Michał Zakliczyński, Wojciech Kosmala

**Affiliations:** 1 Institute of Heart Diseases, Wrocław Medical University, 50-556 Wrocław, Poland; roman.przybylski@umw.edu.pl (R.P.); mateusz.sokolski@umw.edu.pl (M.S.); monika.przewlocka-kosmala@umw.edu.pl (M.P.-K.); michal.zakliczynski@umw.edu.pl (M.Z.); wojciech.kosmala@umw.edu.pl (W.K.); 2 Institute of Heart Diseases, University Clinical Hospital, 50-556 Wrocław, Poland; mateusz.rakowski@usk.wroc.pl (M.R.); jakub.ptak@usk.wroc.pl (J.P.); 3 Pre-Clinical Research Center, Wrocław Medical University, 50-367 Wrocław, Poland; przemyslaw.sarelo@umw.edu.pl; 4 Department of Biomedical Engineering, Faculty of Fundamental Problems of Technology, Wrocław University of Science and Technology, 50-370 Wrocław, Poland

**Keywords:** left atrium, technique of orthotopic heart transplantation, strain

## Abstract

**Background/Objectives**: Currently, the most popular techniques for orthotopic heart transplantation (OHTx) are bicaval and total OHTx. Although bicaval OHTx has shown advantages over the biatrial approach, comparisons between bicaval and total OHTx reain limited. To compare the functional and morphological characteristics of the left atrium (LA) in patients after bicaval and total OHTx. **Methods**: Sixty-six patients (age 51.2 ± 10.5 years) after total OHTx (33 patients) and bicaval OHTx (33 patients) were included in this case–control study. Recipients were matched for sex, age, and time from transplantation and absence of severe graft rejection based on routine endomyocardial biopsies (EMB) performed during follow-up. Echocardiography included standard measurements along with a speckle-tracking assessment of LA strain. **Results**: Compared with the bicaval OHTx, the total OHTx group showed higher atrial mitral inflow velocity, resulting in a lower E/A ratio, lower LA volume index, and higher LA emptying fraction. Both the reservoir and contraction components of LA function, as assessed by LA deformation, were found to show more favorable profiles in the total OHTx group than in the bicaval group (26.5 ± 6.9 vs. 17.4 ± 4.7, *p* < 0.001 and 14.8 ± 5.8 vs. 6.0 ± 4.5, *p* < 0.001, respectively). Multivariable analysis identified surgical technique, left ventricular global longitudinal strain, and the presence of diabetes in the recipient as independent determinants of LA strain. **Conclusions**: Total OHTx is associated with better LA morphology and function than bicaval OHTx. This may provide better conditions for LA-LV coupling in transplanted hearts and contribute to a more stable electrophysiological environment in atrial tissue.

## 1. Introduction

**Figure 1:**
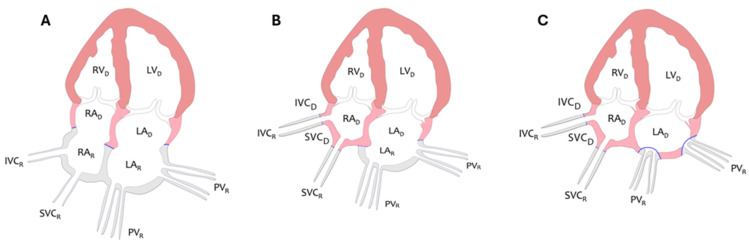
Diagram illustrating principles of biatrial (**A**), bicaval (**B**), and total technique (**C**) of OHTx from an atrial perspective. LA, left atrium; LV, left ventricle; RA, right atrium; RV, right ventricle; IVC, inferior vena cava; SVC, superior vena cava; PV, pulmonary veins; D, donor; R, recipient. Pink and orange color corresponds to the donor’s heart, gray color to the recipient’s heart, and blue color indicates the anastomosis between the donor and recipient’s heart.

Orthotopic heart transplantation (OHTx) remains the preferred treatment for end-stage heart failure in the absence of contraindications. Over the past decades, the biatrial technique for OHTx has been replaced by bicaval or total OHTx. In contrast to the biatrial approach, which preserves a considerable amount of the recipient’s left atrial (LA) and right atrial tissue, the bicaval and total OHTx techniques preserve the donor’s right atrium (RA), maintaining its geometric and conduction properties [1,2]. The differences between bicaval and total OHTx lie in the way of LA connection to the recipient’s pulmonary veins. Bicaval OHTx preserves part of the recipient’s LA as the posterior wall with the pulmonary vein ostia, while total OHTx involves the complete excision of the recipient’s LA, with the recipient’s pulmonary veins integrated with the donor’s LA via two small cuffs. Consequently, the architecture of the donor’s LA remains preserved (Figure 1).

The bicaval technique is the most commonly used OHtx technique in recent decades. The total OHTx is less frequently performed in clinical practice. The major limitation for more widespread use of total OHTx is the increasing combined heart and lung harvest for different recipients when the LA posterior wall may be damaged during the harvesting procedure.

Numerous studies have demonstrated the superiority of bicaval OHTx over the biatrial approach in clinical outcomes, such as less need for pacemaker implantation, fewer incidences of atrial fibrillation and flutter, lower rate of systemic embolism, and less frequent development of significant tricuspid regurgitation [3,4,5,6,7,8,9]. The occurrence of atrial fibrillation in the early post-transplant period (30 days) was reported to be 6.3–17.4% and was the lowest with the total OHTx technique compared with other transplant methods [10]. In addition to LA enlargement, LA functional abnormalities have been proven to contribute to the development of supraventricular tachyarrhythmias and heart failure [11,12,13].

While existing evidence indicates that LA morphology and function are better preserved in heart transplant recipients subjected to the bicaval than biatrial OHTx [10,14,15,16], analogous data comparing the total and bicaval OHTx are scarce. In view of the more homogenous LA structure after the total OHTx, including healthier donor tissue than after the bicaval OHTx, the former approach might be expected to be associated with a more physiological LA morpho-functional remodeling, thus providing less substrate for rhythm disturbances.

To test this hypothesis, we sought to compare LA morphological and functional characteristics between heart transplant recipients treated with the bicaval and total OHTx.

## 2. Materials and Methods

### 2.1. Patients and Study Design

**Figure 2:**
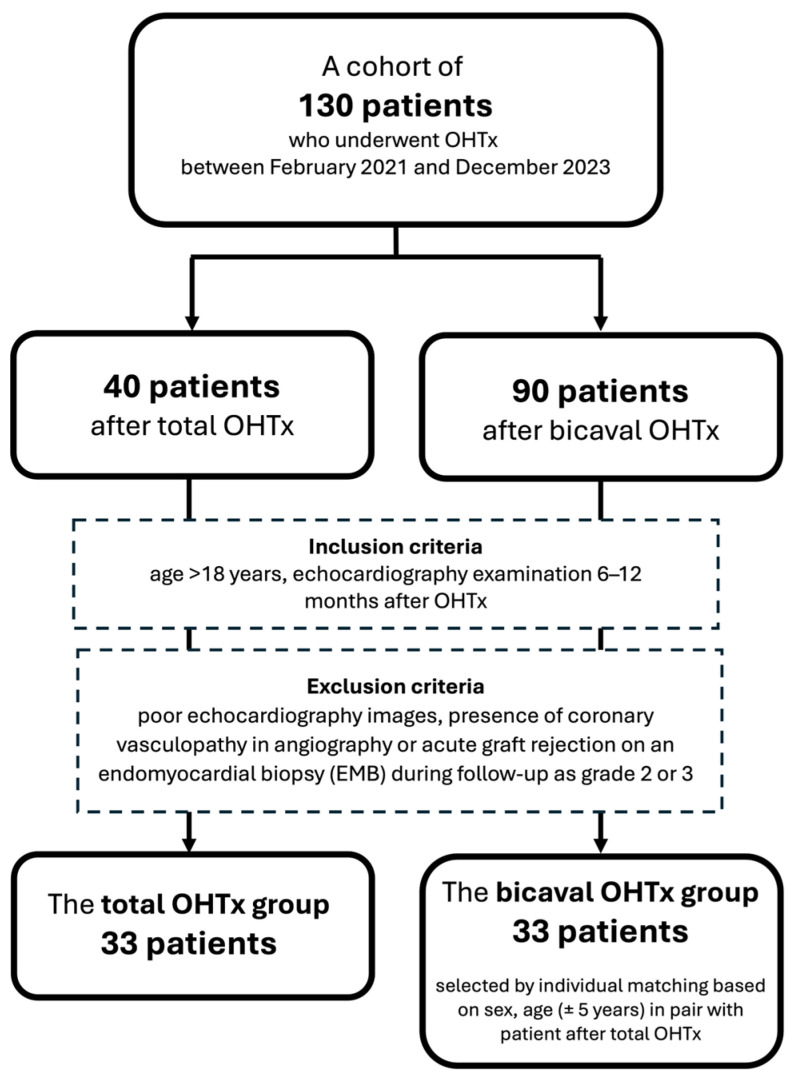
Patient selection for this study.

The population of this retrospective case–control study was derived from a cohort of 130 patients who underwent OHTx between February 2021 and December 2023 at Wroclaw University Hospital. Inclusion criteria encompassed age >18 years and echocardiography examination performed after 6 to 12 months after OHTx. Exclusion criteria were poor echocardiography images, presence of coronary vasculopathy in angiography, or acute graft rejection on an endomyocardial biopsy (EMB) during follow-up as grade 2 or 3 [17]. Of the 40 patients operated on with the total OHTx technique, 33 patients met the inclusion criteria and constituted the total OHTx group. Of the remaining patients who underwent the bicaval OHTx procedure, a compatible subset of 33 subjects was selected by individual matching based on sex and age (±5 years), forming the bicaval group (Figure 2). This study was conducted in accordance with the Declaration of Helsinki and approved by the local Bioethics Committee.

An echocardiographic examination was performed during the routine hospitalization for endomyocardial biopsy (EMB) in all patients enrolled in the study. Likewise, routine laboratory parameters such as hemoglobin concentration, C-reactive protein, plasma creatinine, estimated glomerular filtration rate (eGFR), troponin, and NT-proBNP were assessed on the day of echocardiography. Additionally, data were obtained on comorbidities such as diabetes and hypertension, mean pulmonary pressure measured invasively in heart recipients before OHTx, the occurrence of atrial arrhythmias after OHTx, and post-transplant pharmacotherapy. Donor and cold ischemia time during the transplantation procedure were also recorded.

The occurrence of cardiac arrhythmias was assessed by ECG telemetry during the first 7 days after OHTx, 24 h ECG monitoring before being discharged after transplantation, and during routine hospitalization for EMB in the event of any arrhythmias in the resting ECG.

### 2.2. Echocardiography Examination

Echocardiographic imaging was performed using standard equipment (Vivid e9, General Electric Medical Systems, Horten, Norway), and imaging data were analyzed offline after being saved on a secure server. Cardiac dimensions and wall thicknesses were measured according to the recommendations of the European Association of Cardiovascular Imaging and the American Society of Echocardiography [18]. LV and LA volumes were evaluated by the biplane Simpson method. LV end-diastolic and end-systolic volumes in the apical 4- and 2-chamber views were used for calculation of ejection fraction. LA volume index was obtained by indexing LA maximal volume-to-body surface area. LA emptying fraction was calculated as (LA maximum volume − LA minimum volume) × 100/LA maximum volume. Right ventricular longitudinal systolic function was assessed by tricuspid annular plane systolic excursion in the apical 4-chamber view using M-mode echocardiography. Right atrial area (RAa) was measured in the apical 4-chamber view at end-systole. Peak tricuspid regurgitation velocity was evaluated in the apical 4-chamber view or the parasternal short axis view using continuous wave Doppler.

LV inflow parameters, including peak early (E) and late or atrial (A) diastolic flow velocities, and deceleration time of the early diastolic wave were assessed from the apical 4-chamber view using a pulsed-wave Doppler. The sample volume was placed between the tips of the mitral leaflets.

The pulsed-wave tissue Doppler was used to evaluate peak early diastolic tissue velocity of the mitral annulus from either the lateral or septal side. Then, the ratio of E to the average e’ velocity from both parts of the mitral annulus was calculated.

**Figure 3:**
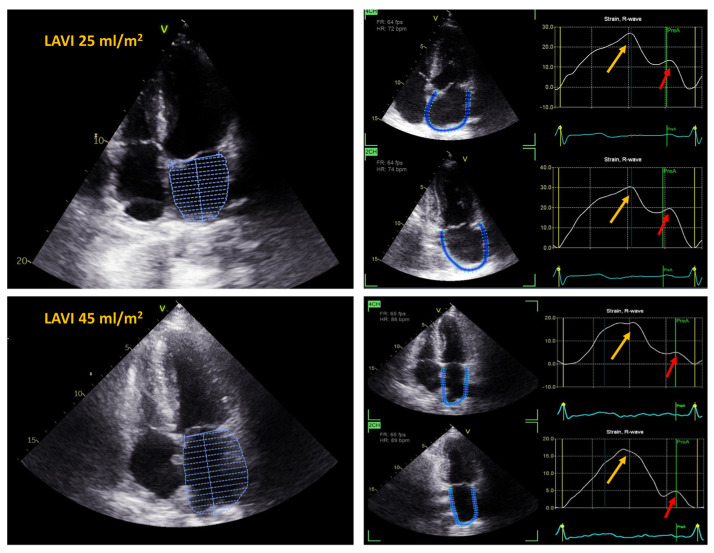
Left atrial strain and left atrial volume index (LAVI) in a patient after total OHTx (**upper panel**) and in a patient after bicaval OHTx (**lower panel**). Yellow arrows indicate peak atrial longitudinal strain (PALS), red arrows indicate peak atrial contraction strain (PACS); 4CH—4 chamber view; 2CH—2 chamber view.

The assessment of LA deformation was performed using the semiautomated 2-dimensional speckle-tracking technique (Echopac PC version 204, General Electric Medical Systems, Horten, Norway). The analysis encompassed all segments of LA from the apical 4- and 2-chamber views recorded with a temporal resolution of 60 to 90 frames/s. The onset of the QRS complex was used as the reference point for strain curves. The endocardial border was manually defined using a 3-point click method, and the appropriate wall thickness was selected. Subsequently, the software tracked the motion of acoustic markers, automatically generated and displayed LA deformation curves, and computed the average strain in each apical view The peak atrial value of the longitudinal strain measured during the LV systole corresponding to the LA reservoir function was calculated as the peak atrial longitudinal strain (PALS). The value of the strain at the onset of the P-wave in the electrocardiogram, with respect to the atrial contractile function, was measured as the peak atrial contractile strain (PACS) (Figure 3). The final values of the LA strain were the averages from both apical views.

Based on PALS and the value of E and E/e’, the LA stiffness index was calculated as E/e’/PALS.

Using the same 2-dimensional speckle-tracking method as for the LA strain, LV longitudinal deformation was evaluated in the apical 4-, 2-, and 3-chamber views. In each view, the greatest negative values on the strain curve during LV systole were measured. Global longitudinal strain (LV GLS) was calculated as the average from all 3 views. Finally, the LV and LA strains were reported as absolute values.

### 2.3. Statistical Analysis

Continuous variables with a normal distribution were expressed as means and standard deviations (SD) and compared using Student’s *t*-test. Variables without a normal distribution were reported as medians with interquartile ranges (IQRs) and analyzed using the Mann–Whitney U-test. Qualitative variables were presented as numbers and percentages, with comparisons performed using the chi-squared test, incorporating the Yates correction when appropriate. The relationships between parameters were examined using the Pearson correlation coefficient for parametric variables and the Spearman correlation for non-parametric ones. LA morphology (left atrial volume index [LAVI]) and function (PALS, PACS) with demographics, concomitant diseases, OHTx surgical technique, and LV function. A series of stepwise multiple linear regression models was developed to identify the independent determinants of PALS, PACS, and LAVI in heart transplant recipients. The components of these models were selected based on anticipated and univariate association. The variables were placed in the models when the univariate analysis *p*-value was less than 0.2.

To clarify the nature of the relationship between the OHTx technique and LA function and morphology, mediation analysis was employed [19], testing a putative causal relation between heart transplant technique and PALS (a marker of LA function) via LAVI (a marker of LA morphology) as a mediator.

Statistical analyses were carried out with standard statistical software (Statistica version 13, TIBCO Software Inc., Palo Alto, CA, USA) and GraphPad Prism 10 (Dotmatics, Boston, MA, USA), in which a *p*-value of 0.05 was considered to be statistically significant.

## 3. Results

### 3.1. Demographic and Clinical Characteristics

Among the 66 transplant recipients included in the study (age 51.2 ± 10.5 years, 85% male), ischemic heart disease was the reason for OHTx in 25 patients, dilated cardiomyopathy in 28 patients, congenital heart disease in 5 patients, post-inflammatory disease in 5 patients, and an unknown cause in 3 patients. Before OHTx, five patients received mechanical circulatory support by left ventricular assist therapy with HeartMate 3.

The demographic, laboratory, and clinical profiles of the bicaval OHTx and total OHTx groups are presented in Table 1.

There were no differences between the groups in the demographic and laboratory parameters.

The prescription of antihypertensive medications after OHTx was similar in both groups. The immunosuppressive regimen was based on tacrolimus and mycophenolate mofetil in all enrollees. All patients included in the study were administered similar doses of steroids during the 6 months after OHTx. In none of the studied patients, atrial fibrillation was reported in an early post-transplant period or during hospitalizations for routine EMB.

### 3.2. Echocardiographic Characteristics

**Figure 4:**
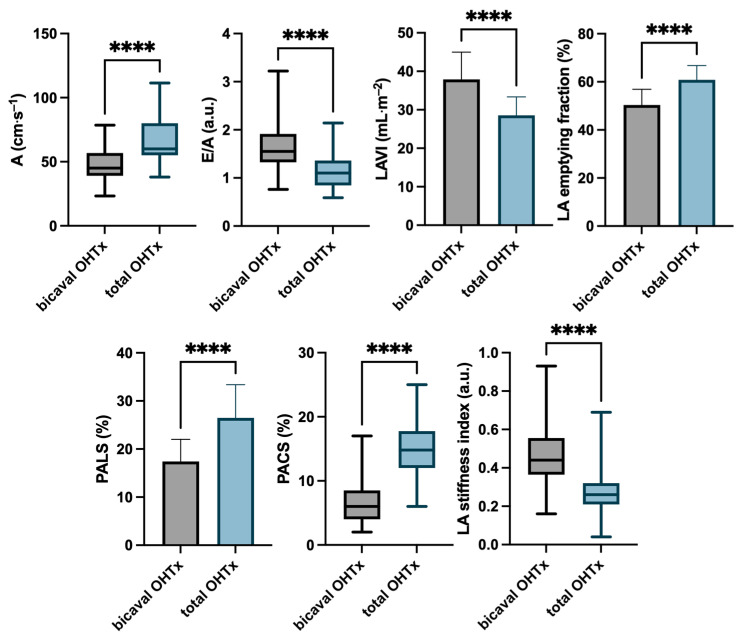
Echocardiographic characteristics of the studied groups according to the type of surgical technique of orthotopic heart transplantation. **** *p* < 0.001.

Echocardiographic parameters in both groups are displayed in Table 2 and Figure 4.

No differences were found between the groups in LV dimensions, ejection fraction, and GLS.

In the analysis of Doppler parameters, differences in mitral inflow were noted. In the bicaval OHTx group, the A-wave velocity was lower, and the E/A ratio was higher than in the total OHTx group.

LAVI was found to be significantly lower in the total OHTx group than in the bicaval OHTx group, with a value below 34 mL·m^−2^, which falls in the upper limit of a normal range in healthy subjects. LA emptying fraction was significantly higher in the total OHTx group compared with the bicaval OHTx group.

PALS, as a marker of reservoir LA function, and PACS, as a marker of LA contraction, were significantly higher in patients after total OHTx compared with bicaval OHTx. Furthermore, the LA stiffness index (E/e’/PALS) was lower in the total OHTx group.

Both OHTx techniques allowed for the preservation of the donor’s RA, and no differences between the groups in the RA area or in the parameters of right ventricular function were demonstrated.

### 3.3. Independent Determinants of LA Characteristics

Univariate associations of PALS, PACS, and LAVI with clinical characteristics are presented in Table 3.

The type of transplantation technique was found to be an independent determinant in multivariable regression analysis models performed separately for LAVI, PALS, and PACS. Additionally, LV GLS was identified as an independent determinant for PALS and diabetes mellitus in the medical history before OHTx for PACS. The results of multivariate regression analyses are shown in Table 4.

**Figure 5:**
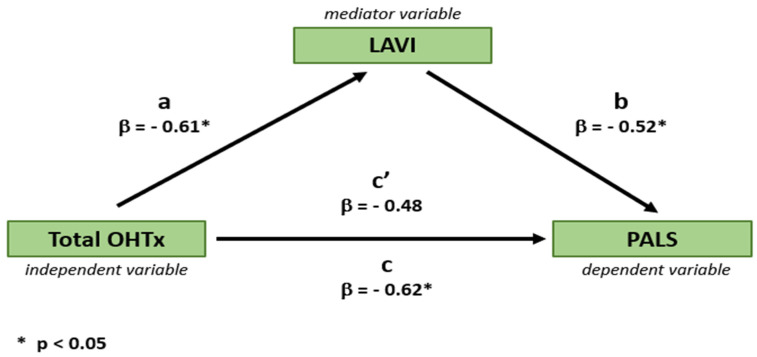
Mediating effect of left atrial size (LAVI) on the relationship between the type of OHTx technique and left atrial function (PALS). β, beta coefficient; a, beta coefficient of OHTx technique predicting LAVI in a univariable regression analysis; b, beta coefficient of LAVI predicting PALS in a univariable regression analysis; c, beta coefficient of OHTx technique predicting PALS in a univariable regression analysis (i.e., without the mediating effect of LAVI); c’, beta coefficient of OHTx technique predicting PALS in a multivariable regression analysis including OHTx technique and LAVI (i.e., with the mediating effect of LAVI).

Regression-based mediation analysis demonstrated that the relationship between the OHTx technique and PALS was partially mediated by LAVI, as evidenced by a small reduction in the variance of PALS, explained by the type of OHTx technique after the inclusion of LAVI to the model (decrease in beta coefficient from 0.66 to 0.61 (Figure 5)).

## 4. Discussion

The major finding of this study is that LA function and morphology are independently associated with the OHTx technique, being better preserved in heart transplant recipients after total OHTx than after bicaval OHTx. This might provide support for the preferred use of the total OHTx technique in clinical practice.

LA plays an important role in cardiac physiology, providing hemodynamic support for the LV and mirroring abnormalities of LV filling. Although the mainstay for the performance of a transplanted heart is ventricular function, the atrial-associated aspects, such as atrial contribution to maintaining adequate atrial–ventricular coupling and the risk of developing atrial pathologies leading to arrhythmias, should not be ignored in transplantation strategies. The evolution of surgical techniques affords an opportunity to improve post-transplant LA anatomy and function. Previous studies showed the superiority of the bicaval over the biatrial technique in terms of anatomy and functionality of the atria [15,16,20,21]. Total OHTx ensures the smallest volume and best preservation of LA function and, consequently, a more physiological perspective for atrial–ventricular interdependence. This may explain the lower prevalence of atrial fibrillation reported in the literature in patients treated with this method [10].

The total OHTx technique is the most LA-oriented approach to heart transplant, offering the largest preservation of the donor atrial tissue. Our study demonstrated the advantages of this technique over the bicaval method, both in terms of LA size and function. LA enlargement found in most patients treated with the bicaval technique is a direct consequence of leaving during this procedure a considerable amount of remodeled recipient atrial tissue. It increases the risk of atrial arrhythmias development and contributes to LA dysfunction. However, our mediation analysis revealed that the worse post-transplant LA function could be only partially attributable to concomitant LA dilatation, and the detrimental impact of bicaval OHTx extends beyond the effect of this technique on LA size.

The analysis of atrial strain allows for a comprehensive assessment of all phases of LA function. Both the reservoir phase, assessed by PALS, and the contraction phase, assessed by PACS, were found to show more favorable profiles in the total OHTx group than in the bicaval group.

The reservoir phase reflects LA diastolic properties, as well as LV function [22,23,24]. We found that LV GLS—a marker of LV longitudinal contractility—was among the independent determinants of PALS, which is consistent with findings in other clinical conditions [25,26,27,28,29,30]. No significant intergroup differences in this parameter, as well as the absence of data on the varying effects of different heart transplantation techniques on LV function, deny the role of impaired LV GLS as a mediator of worse post-transplant LA performance in patients subjected to the bicaval OHTx.

In addition to lower PACS, post-transplant impairment in LA contraction in the bicaval group was observed in mitral inflow Doppler parameters, as evinced by a significantly lower A wave velocity and a more “restrictive” E/A pattern [31]. Ultimately, lower LA volume and better LA reservoir and contraction function lead to a larger LA emptying fraction in the total OHTx subset. The reduced LA contribution to LV filling independent of LV performance, due to the transplant-technique-associated changes in LA structure and function, should be taken into account when evaluating LV diastolic function in patients after bicaval OHTx.

The greater impairment of LA properties in the bicaval OHTx group was also evidenced by the higher LA stiffness index, calculated on the basis of PALS and E/e’ ratio. However, it should be stressed that this parameter, although previously validated in invasive studies [32,33], provides only indirect insight into LA structural characteristics.

Implications. These study findings may support the use of the total OHTx technique in heart transplant surgery. However, to make a stronger recommendation for this approach, more evidence for clinical benefit is required. This poses the need for multicenter studies assessing the impact of various transplant techniques on clinical outcomes, including the occurrence of arrhythmias. On the other hand, the unrestricted use of total OHTx is limited by the inability to simultaneously harvest lungs for another recipient. Therefore, further improvements in lung harvesting techniques to preserve the integrity of the left atrial architecture are needed.

Given the potential risk of extending the pro-arrhythmic milieu in atrial tissue by surgical procedures, assessment of LA function might be considered, in addition to LA size measurement, as part of routine echocardiogram in heart transplant recipients.

The transplanted heart, deprived of physiological autonomic regulation due to its denervation, is more preload-dependent and relies on preserved Frank–Starling mechanisms. In this context, the role of the atria may be crucial in ensuring adequate ventricular filling and maintaining optimal ventricular performance, with possible implications for heart failure development, which, however, needs further studies.

More physiological conditions for LA-LV coupling following total OHTx may facilitate the assessment of LV diastolic function, which might be important in surveying patients for graft rejection.

Limitations. First, the small sample size might have impacted the significance of intergroup comparisons, and the case–control design might have provided a selection bias. Second, the relatively short follow-up without systematic Holter ECG monitoring restricts concluding on the actual association of LA mechanics with atrial arrhythmias.

## 5. Conclusions

The total OHTx technique is associated with more favorable LA morphology and function than bicaval OHTx. This may provide better conditions for LA-LV coupling in transplanted hearts and contribute to a more stable electrophysiological environment in atrial tissue.

## Figures and Tables

**Table 1:** Demographic, laboratory, and clinical characteristics of the studied groups according to the type of surgical technique of heart transplantation.

	Bicaval OHTx	Total OHTx	*p*-Value
	N = 33	N = 33	
Age, years (SD)	51.4 (10.5)	51.0 (10.9)	0.89
Women, %	15	15	
BMI, kg∙m^−2^ (SD)	26.7 (3.8)	25.2 (3.8)	0.15
Heart rate, bpm (SD)	88 (9)	89 (10)	0.79
NT-proBNP, pg∙mL^−1^ (IQR)	602 (317–1245)	1137 (306–2026)	0.50
Troponin, (μg∙L^−1^) (IQR)	7.33 (3.13–14.96)	8.90 (4.13–12.94)	0.54
Hemoglobin, g∙dL^−1^ (SD)	12.9 (1.5)	12.4 (1.7)	0.31
CRP, mg∙L^−1^ (IQR)	2.7 (1.2–11.5)	5.7 (1.6–21.9)	0.32
Creatinine, mg∙dL^−1^ (SD)	1.18 (0.39)	1.24 (0.41)	0.51
eGFR, mL∙min^−1^∙1.73 m^−2^ (SD)	75.1 (22.5)	68.2 (23.7)	0.36
Age of donor, years (SD)	41 (13)	42 (11)	0.81
Cold ischemic time, min (SD)	115 (52)	132 (55)	0.29
Hypertension (%)	48%	33%	0.29
Diabetes mellitus (%)	28%	13%	0.20
Preoperative mean PAP, mmHg (SD)	29.3 (10.4)	27.6 (10.5)	0.62
BB (%)	30%	23%	0.74
ACEI or ARB (%)	42%	30%	0.44
CB (%)	36%	33%	0.79

Continuous variables are given as means and standard deviations (SD) or medians and interquartile ranges (IQR) (25–75th). ACEI, angiotensin-converting enzyme inhibitors; ARB, angiotensin receptor blockers; BB, beta-blocker; BMI, body mass index; bpm beats per minute, CB, calcium blocker; CRP, C-reactive protein; eGFR, estimated glomerular filtration rate; PAP, pulmonary artery pressure; NT-proBNP, N-terminal prohormone of brain natriuretic peptide.

**Table 2:** Echocardiographic characteristics of the studied groups according to the type of surgical technique of heart transplantation.

	Bicaval OHTx	Total OHTx	*p*-Value
	N = 33	N = 33	
LVDd, mm (SD)	48 (8)	46 (8)	0.34
IVDd, mm (SD)	12 (2)	12 (1)	0.59
PWDd, mm (SD)	11 (1)	10 (1)	0.52
LV EF, % (SD)	59 (5)	58 (5)	0.91
LV GLS, % (SD)	15.6 (3.0)	16.6 (2.2)	0.12
CO, L∙min^−1^ (SD)	48 (1.1)	4.2 (1.4)	0.23
E, cm∙s^−1^ (SD)	76 (18)	65 (15)	0.08
A, cm∙s^−1^ (SD)	48 (14)	65 (19)	<0.001
DT, ms (SD)	148 (29)	156 (44)	0.26
E/A (SD)	1.7 (0.5)	1.1 (0.4)	<0.001
e’ lateral, cm∙s^−1^ (SD)	13.0 (3.1)	12.0 (3.4)	0.24
e’ septal, cm∙s^−1^ (SD)	7.8 (2.1)	7.4 (1.5)	0.35
E/e’ (SD)	7.3 (2.2)	7.0 (3.1)	0.54
LAVI, mL∙m^−2^ (SD)	37.9 (7.1)	28.6 (4.8)	<0.001
LA emptying fraction, % (SD)	50.4 (6.5)	60.8 (6)	<0.001
PALS, % (SD)	17.4 (4.7)	26.5 (6.9)	<0.001
PACS, % (SD)	6.0 (4.5)	14.8 (5.8)	<0.001
LA stiffness index (SD)	0.44 (0.19)	0.26 (0.24)	<0.001
TR velocity, m∙s^−1^ (SD)	2.2 (0.4)	2.3 (0.4)	0.46
TAPSE, mm (SD)	17 (2)	17 (2)	0.96
RAa, cm^2^ (SD)	15.0 (1)	15.0 (1.5)	0.98

A, atrial wave velocity of mitral inflow; E, early wave velocity of mitral inflow; e’, early peak velocity of mitral anulus; CO, cardiac output; DT, deceleration time; EF, ejection fraction; GLS, global longitudinal strain; IVDd, intraventricular end-diastolic diameter; LA, left atrium; LAVI, left atrium volume index; LV, left ventricle; LVDd, left ventricular end-diastolic dimeter; OHTx, orthotopic heart transplantation; PACS, peak atrial contraction strain; PALS, peak atrial longitudinal strain; PWDd, posterior wall end-diastolic diameter; RAa, right atrium area; TAPSE, tricuspid annular plane systolic excursion; TR, tricuspid regurgitation.

**Table 3:** Associations of PALS, PACS, and LAVI with echocardiographic and clinical parameters: univariate analysis.

	PALS		PACS		LAVI	
	R	*p*	R	*p*	R	*p*
BMI	−0.36	<0.001	−0.34	0.04	0.24	0.16
Hypertension	−0.45	0.008	−0.54	0.001	0.39	0.02
Diabetes mellitus	−0.48	0.004	−0.48	0.004	0.36	0.03
Age of donor	−0.05	0.79	0.09	0.62	0.04	0.81
Cold ischemia time	0.23	0.19	0.17	0.34	−0.23	0.20
Total OHTx technique	0.69	<0.001	0.82	<0.001	−0.81	<0.001
LAVI	−0.52	0.002	−0.62	<0.001		
LV GLS	0.39	0.02	0.33	0.05	−0.16	0.37

BMI, body mass index; GLS, global longitudinal strain; LAVI, left atrial volume index; LV, left ventricle; OHTx, orthotopic heart transplantation; PALS, peak atrial longitudinal strain; PACS, peak atrial contraction strain.

**Table 4:** Associations of PALS, PACS, and LAVI with echocardiographic and clinical parameters: multivariable analysis.

	Model for PALS R^2^ = 0.53			Model for PACS R^2^ = 0.58			Model for LAVI R^2^ = 0.43		
	β	SE	*P*	β	SE	*p*	β	SE	*p*
Total OHTx technique	0.50	0.10	<0.001	0.66	0.09	<0.001	−0.56	0.11	<0.001
LV GLS	0.22	0.10	0.025	0.02	0.09	0.84	0.03	0.11	0.76
BMI	−0.10	0.10	0.32	−0.07	0.09	0.46	0.04	0.11	0.73
Diabetes mellitus	−0.24	0.11	0.026	−0.19	0.10	0.08	0.12	0.11	0.29
Hypertension	−0.03	0.11	0.78	−0.12	0.10	0.24	0.13	0.12	0.27

BMI, body mass index; GLS, global longitudinal strain; LAVI, left atrial volume index; LV, left ventricle; OHTx, orthotopic heart transplantation; PALS, peak atrial longitudinal strain; PACS, peak atrial contraction strain.

## Data Availability

The data that support the findings of this study are available from the corresponding author upon reasonable request.

## Abbreviations

eGFR	estimated glomerular filtration rate
EMB	endomyocardial biopsy
GLS	global longitudinal strain
LA	left atrium
LAVI	left atrial volume index
LV	left ventricle
OHTx	orthotopic heart transplantation
RA	right atrium
PACS	peak atrial contraction strain
PALS	peak atrial longitudinal strain
